# Inflammatory and Prothrombotic Biomarkers Contribute to the Persistence of Sequelae in Recovered COVID-19 Patients

**DOI:** 10.3390/ijms242417468

**Published:** 2023-12-14

**Authors:** Nallely Garcia-Larragoiti, Alan Cano-Mendez, Yeny Jimenez-Vega, Mercedes Trujillo, Patricia Guzman-Cancino, Yesenia Ambriz-Murillo, Martha Eva Viveros-Sandoval

**Affiliations:** 1 División de Estudios de Posgrado, Facultad de Ciencias Médicas y Biológicas “Dr. Ignacio Chávez”, Universidad Michoacana de San Nicolás de Hidalgo, Morelia 58060, Michoacán, Mexico; 2 Centro Multidisciplinario de Estudios en Biotecnología, Facultad de Medicina Veterinaria y Zootecnia, Universidad Michoacana de San Nicolás de Hidalgo, Morelia 58060, Michoacán, Mexico; 3 Hospital Regional de Morelia ISSSTE, Morelia 58300, Michoacán, Mexico

**Keywords:** long COVID, metabolic syndrome, inflammation markers, prothrombotic biomarkers, immunothrombosis

## Abstract

The presence of long COVID (LC) following SARS-CoV-2 infection is a common condition that affects the quality of life of patients and represents a diagnostic challenge due to the diversity of symptoms that may coexist. We still do not have accurate information regarding the pathophysiological pathways that generate the presence of LC, and so it is important to know the inflammatory and immunothrombotic biomarker profiles and their implications in order to characterize risk subgroups and establish early therapeutic strategies. We performed the determination of inflammatory and immunothrombotic biomarkers in volunteers with previous diagnoses of SARS-CoV-2. The inflammatory biomarkers were analyzed in plasma by flow cytometry, and we analyzed the von Willebrand factor (vWF) in the plasma samples using ELISA. The clinical variables and the presence or absence of long COVID symptoms were then analyzed. IL-6, sCD40L, p-Selectin, PSGL-1, PAI-1, tPA, D-Dimer, TF, and Factor IX levels were elevated in the groups with LC, especially in the subgroup of patients with metabolic syndrome (MetS). VWF levels were found to be increased in patients with sequelae and MetS. Our results confirmed the persistence of an active immunothrombotic state, and so it is important to identify the population at risk in order to provide adequate clinical follow-up.

## 1. Introduction

The coronavirus disease 2019 (COVID-19), which is caused by infection with severe acute respiratory syndrome coronavirus 2 (SARS-CoV-2), is characterized by a broad spectrum of clinical manifestations [1]. A significant number of COVID-19 survivors have reported the persistence of symptoms 12 weeks or more after the acute infection process that continues even 8 months after the acute phase of the disease [2,3,4,5]. This has been defined as post-acute COVID-19 syndrome (PACS) or long COVID (LC) [6]. LC has been associated with a wide range of symptoms, affecting people of all ages and both genders [7]. LC symptoms include respiratory, neurologic, cardiovascular, gastrointestinal, psychosocial, and metabolic affections [8,9]. Most cases do not require hospital attention; however, LC has a direct impact on the lifestyles of the people who experience the condition [10,11]. Several pathophysiological mechanisms have been proposed to explain the clinical manifestations observed in LC; however, the enigma about the etiology of the disease remains poorly described [2,7,12]. It has been reported that there is a relationship between the persistence of symptoms associated with LC and the presence of viral RNA or the spike (S) protein of the SARS-CoV-2 virus in the plasma samples of affected patients, even 12 months after the infectious process has concluded [13,14]. The activation by these pathogen-associated molecular patterns (PAMPs) of immune cells such as macrophages, neutrophils, and adaptative immune cells such as T (CD4+) and C (CD8+) has been reported [14,15,16]. Immune cells release inflammatory cytokines such as interleukin 6 (IL-6), interleukin 8 (IL-8), interleukin 17 (IL-17), interferons (INF), chemokine 8 (CXCL8), chemokine 9 (CXCL9), and chemokine 10 (CXCL10), as well as the induction of the expression of adhesion proteins such as P-selectin (P-sel), E-selectin (E-sel), intercellular adhesion molecule-1 (ICAM-1,) and vascular cell adhesion molecule-1 (VCAM-1), which are associated with the inflammatory process to which the persistence of symptomatology observed in LC is related [14,15,16]. In addition, the activation of memory B lymphocytes, subsequent antibody release, and complement activation have also been linked to the active inflammatory process in LC subjects [16,17]. Inflammatory and coagulation processes are closely linked, and this process is known as thromboinflammation. It has been described that there is an immunothrombotic process in patients with LC [15]. The inflammatory phenotype of neutrophils observed in LC and the subsequent formation of neutrophil extracellular traps (NETs) leads to the activation of surrounding platelets, favoring the establishment of a procoagulant state [18,19]. In addition, the release of platelet microvesicles, rich in phosphatidylserine (PS), induces the activation of the coagulation process, characterized by elevated thrombin concentrations and leading to fibrin formation. This is observed by finding elevated concentrations of fibrin degradation products and dimer d complexes in patients with LC [20]. Endotheliopathy induced by the immunothrombotic process leads to the release of the von Willebrand factor (vWF) from endothelial cells. vWF is a highly thrombogenic protein, and dysregulation of the vFW-ADAMST-13 axis has been associated with the persistence of symptoms in subjects who have recovered from COVID-19 [21,22]. An association between metabolic syndrome (MetS) and LC in subjects recovered from infection has been reported [23]. This is related to the inflammatory stage induced by MetS and the potentiation of the inflammatory state as a consequence of the viral infection [24]. However, the mechanisms linking these entities remain unclear. On the other hand, MetS is a syndrome with a high prevalence in the Latino population, including Mexicans, given their sociodemographic characteristics, lifestyles, and diets [25]. The inflammatory process that this condition represents could increase the risk of suffering from LC in a population affected by the viral infection. The main objective of this study was to assess the immunothrombotic profiles of subjects with COVID-19 and correlate them with the presence of LC, as well as to study the relevance of chronic inflammatory and immune dysregulation and their associations with LC.

## 2. Results

Sixty-eight individuals with diagnosis of COVID-19 in a period of three months to within a year were included. Seventy percent of the participants were female, and all of them had completed their vaccination schedules against SARS-CoV-2. All participants were older than 18 years. The participants were categorized into three groups based on the presence or absence of symptoms consistent with LC and a positive diagnosis of metabolic syndrome. Samples from 23 healthy donors were included as a control group. The clinical and demographic characteristics of the study population are shown in Table 1.

Based on the most common symptoms reported by individuals with LC, a questionnaire was administered to each of the participants, where the symptoms that were most frequently reported included headaches and anxiety, and sore throats and arrhythmia were the most common symptoms that occurred in patients with LC. The complete results are shown in Table 2.

### 2.1. Immunothrombotic Biomarkers

**Figure 1:**
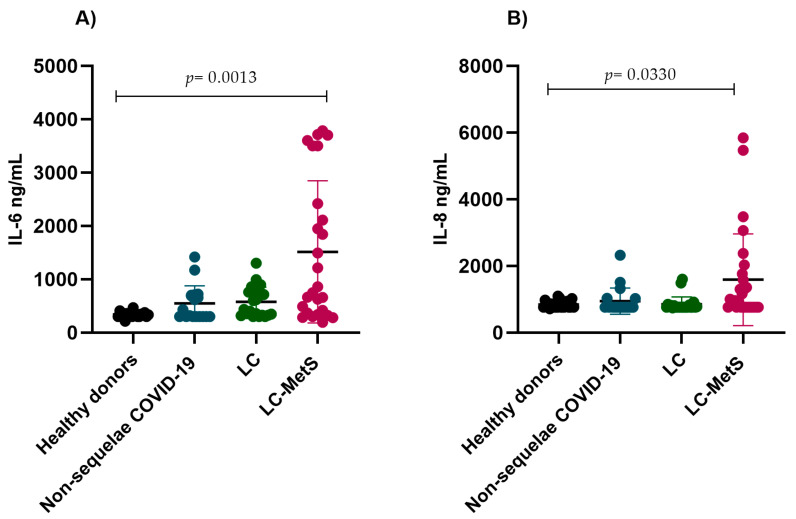
Comparison of the plasma concentrations of IL-6 and IL-8 in the study groups. (**A**) The non-sequelae COVID-19 and LC groups showed similar IL-6 concentrations. The plasma levels of IL-6 increased in the LC-MetS group (Kruskal–Wallis *p* = 0.0013). (**B**) No significant differences were found in the concentrations of IL-8 among the study groups (Kruskal–Wallis *p* = 0.0330).

Recently, the presence of thromboinflammation biomarkers has been associated with post-acute COVID-19 sequelae. To assess the persistence of vascular damage, endothelial dysfunction, and coagulation abnormalities in individuals with and without post-acute COVID-19 sequelae, the circulating concentrations of immunothrombotic biomarkers were analyzed in the plasma samples taken from the participants and those from the group of healthy donors. Our results demonstrated increased plasma IL-6 concentrations in the group of COVID-infected individuals without sequelae and in those with LC and LC-MetS when compared to the healthy donor group. The increases were significantly higher in the LC-MetS group (*p* = 0.0013) (Figure 1A). Although there was a trend towards increased levels of IL-8 in the LC-MetS group, the differences were not significant when comparing between groups (*p* = 0.0623) (Figure 1B).

**Figure 2:**
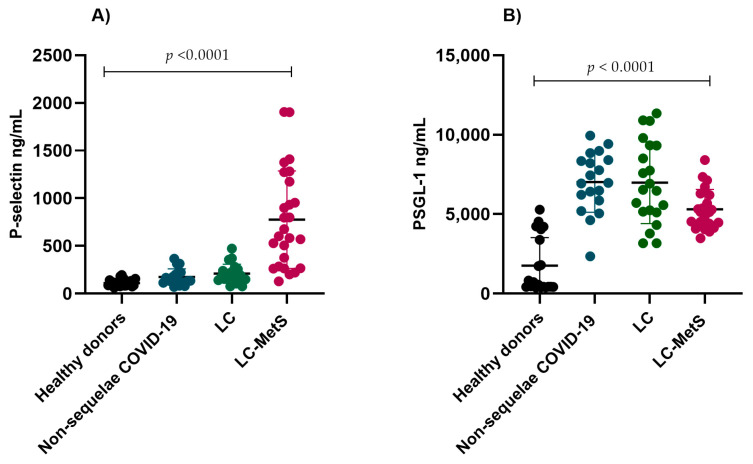
Plasmatic concentrations of P-selectin and PSGL-1 in the study groups. (**A**) There were progressive increases in the plasma levels of p-selectin in all three experimental groups compared to the healthy donor group (Kruskal–Wallis *p* = 0.0001). (**B**) The plasma concentrations of PSGL-1 were significantly elevated in the study groups compared to the control group (Kruskal–Wallis *p* < 0.0001).

When analyzing the plasma concentrations of P-selectin among the groups, we observed increases in the experimental groups compared to the healthy donor group, with the LC-MetS group showing significantly higher concentrations than the other groups (*p* < 0.0001) (Figure 2A). A similar pattern was observed for PSGL-1. The groups with COVID infection without sequelae, LC, and LC-MetS exhibited significantly higher levels than the healthy donor group (*p* < 0.0001) (Figure 2B).

**Figure 3:**
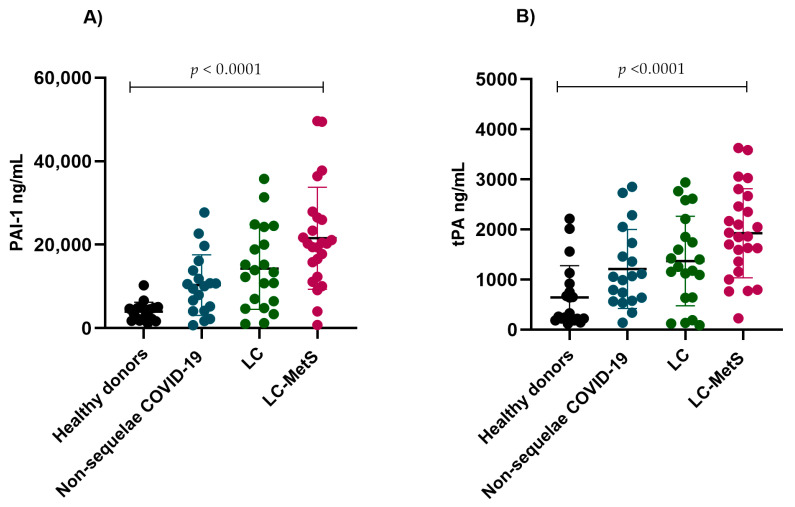
Comparison of the plasma concentrations of PAI-1 and tPA in the study groups. (**A**) The non-sequelae COVID-19, LC, and LC-MetS groups showed significant increases in PAI-I levels compared to the healthy donor group (Kruskal–Wallis *p* < 0.0001). (**B**) A similar pattern was observed for tPA concentrations, where the LC-MetS group had the highest plasma levels compared to the other groups (Kruskal–Wallis *p* < 0.0001).

Given that elevated levels of PAI-1 have been documented as a crucial indicator of endothelial dysfunction in severe COVID-19 patients, we decided to assess its behavior in individuals experiencing post-COVID syndrome. Increased plasma concentrations of PAI-1 were detected in our experimental groups (*p* < 0.0001) (Figure 3A). We also evaluated tPA concentrations and observed that, similar to PAI-1, the plasma concentrations were significantly elevated in the LC and LC-MetS groups (*p* < 0.0001) (Figure 3B).

**Figure 4:**
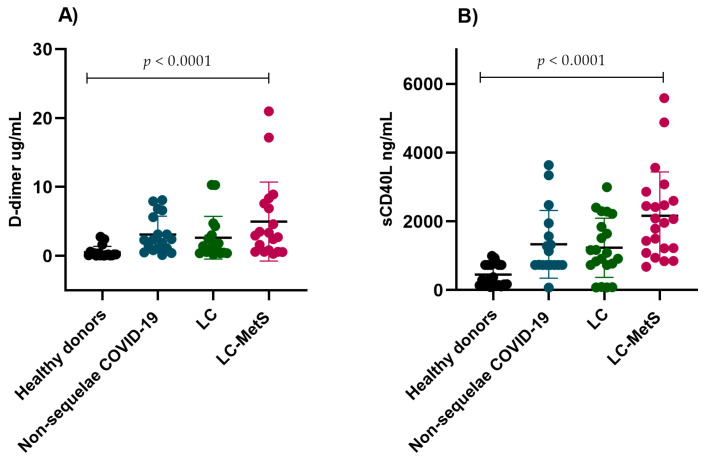
Plasmatic concentrations of D-dimer and sCD40L in the study groups. (**A**) The non-sequelae COVID-19, LC, and LC-MetS groups showed significant increases in D-D levels compared to the healthy donor group (Kruskal–Wallis *p* < 0.0001). (**B**) The plasma concentrations of sCD40L were found to be higher in the experimental groups compared to the healthy donor group (Kruskal–Wallis *p* < 0.0001).

It has been reported that an increase in D-dimer concentrations is correlated with an increase in mortality in COVID-19 patients. However, there is limited information about its correlation with abnormalities in individuals with LC. For this reason, we evaluated the plasma concentrations of D-D in our study groups. Interestingly, the LC and non-sequelae COVID-19 groups showed similar elevated levels compared to the healthy donor group. The most significant difference was observed in the LC-MetS group, where D-D plasma levels were significantly higher than they were in the rest of the groups (*p* < 0.0001) (Figure 4A). We also evaluated CD40L, obtaining similar results, with the presence of elevated concentrations in all three experimental groups being significant when compared to the control group (*p* < 0.0001) (Figure 4B).

**Figure 5:**
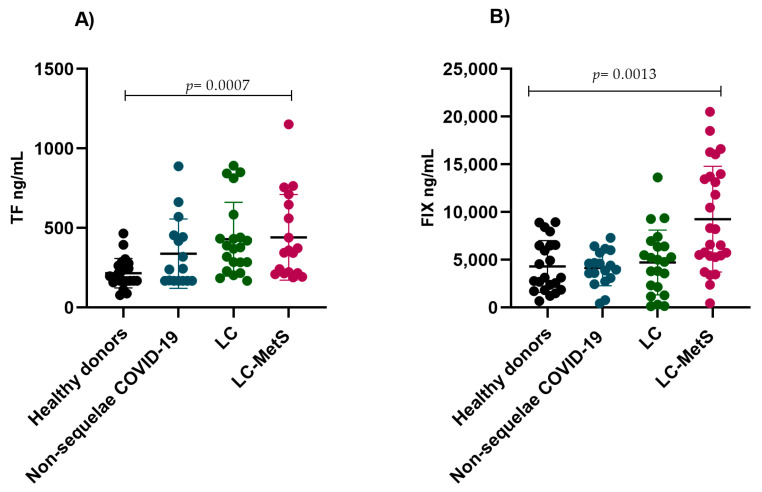
Comparison of the plasma concentrations of TF and FIX in the study groups. (**A**) The non-sequelae COVID-19, LC, and LC-MetS groups showed significant increases in TF levels compared to the healthy donor group (Kruskal–Wallis *p* = 0.0007). (**B**) A similar pattern was observed for tPA concentrations, where the LC-MetS group had the highest plasma levels compared to the other groups (Kruskal–Wallis *p* = 0.0013).

Finally, we analyzed the expression of tissue factor, which is an initiator of the extrinsic coagulation pathway. Previous reports have concluded that elevated concentrations of tissue factor lead to thrombotic complications during SARS-CoV-2 infection, and so we decided to include it in the panel of markers to be assessed in our experimental groups. We observed that regardless of the presence of sequelae or metabolic syndrome, TF levels were significantly higher in all three study groups compared to the healthy donor group (*p* = 0.0007) (Figure 5A). The same trend was observed when analyzing FIX, where the increases in plasma concentrations were greater in the LC-Mets group (*p* = 0.0013) (Figure 5B).

### 2.2. Von Willebrand Factor

**Figure 6:**
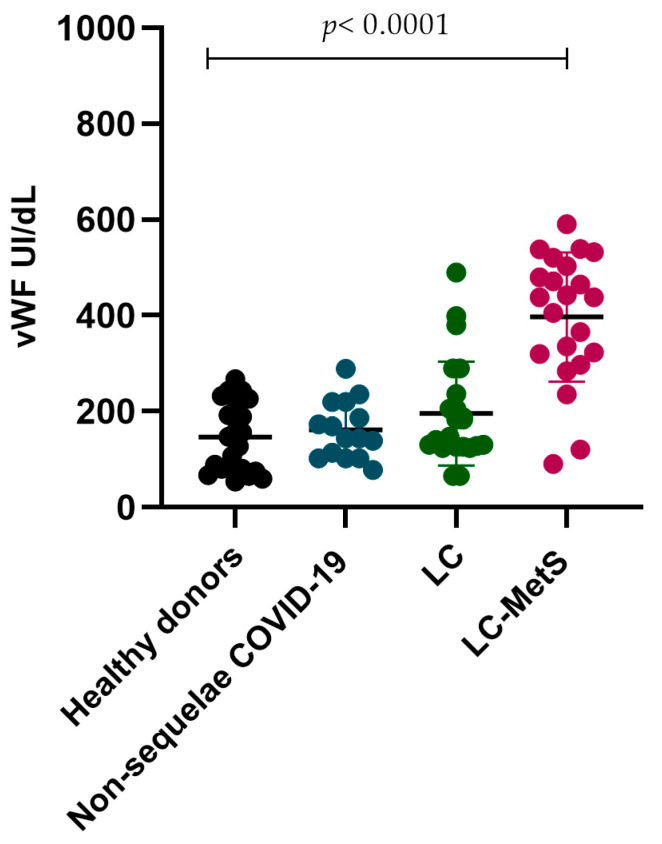
Comparison of the vWF concentrations in the different study groups. The healthy donor group and the non-sequelae COVID-19 group showed similar concentrations of vWF, while the LC and LC-MetS groups exhibited significant increases in the plasma concentrations of vWF (Kruskal–Wallis *p* < 0.0001).

In previous studies, it has been demonstrated that a significant percentage of patients infected with SARS-CoV-2 develop a severe form of the infection and die. Factors such as advanced age, obesity, hypertension, diabetes, and underlying cardiovascular diseases increase the risk of mortality. For this reason, we decided to evaluate the concentrations of the von Willebrand factor in our study groups, as it has shown to be a potential biomarker that helps identify patients with high thrombotic risk. Although our study population focused on individuals who had recovered from COVID-19, our results showed that even after recovering from infection, vWF levels remained elevated in LC and LC-Mets groups compared to the individuals who did not develop sequelae from the disease (*p* < 0.0001) (Figure 6).

## 3. Discussion

The persistence of symptoms after COVID disease is one of the major current challenges for the healthcare system due to the risk it represents to the health of individuals affected by the physical and psychological symptoms associated with the infection, as well as the social, economic, and personal implications it represents. Therefore, it is of great importance to understand the correlation that exists between age, sex, chronic inflammation processes, autoimmune diseases, and the development of this syndrome in order to provide personalized medical care and identify the groups at higher risk of facing post-COVID sequelae in the population.

Hence, the main objective of this research was to provide evidence that inflammatory and prothrombotic biomarkers are associated with LC. One of the most important cytokines in the development of an inflammatory process is IL-6, which plays a crucial role in the evolution and enhancement of acute inflammatory conditions. Elevated IL-6 levels have been significantly linked to unfavorable clinical outcomes in COVID-19, such as the development of acute respiratory distress syndrome (ARDS) and mortality. In addition, patients with severe COVID-19 infection have shown higher levels of IL-6 in their plasma samples compared to those with less severe cases of the disease [26]. An increase in IL-6 levels not only occurs in the period of acute COVID-19 infection but is also one of the most critical factors contributing to post-COVID-19 syndrome. In this study, we demonstrated that increased IL-6 was associated with LC, and we found elevated plasma levels of IL-6 in the non-sequalae LC and de LC groups and significantly higher levels in the LC-MetS group. In another study, Yin J et al. [27] demonstrated that increased IL-6 levels are associated with LC. They found that serum levels of IL-6 were significantly elevated in patients after COVID-19 infection, whether in the acute or prolonged phase of the disease. Although there is evidence that the presence of patients with metabolic comorbidities have greater risks of presenting with severe COVID, there is not much information about patients with MetS who present with LC. These pathophysiological alterations could be contributing to accelerated inflammatory symptoms, which could lead to aggravation of the underlying pathology and enhance the manifestation of symptoms associated with LC. In contrast, IL-8 is known for being one of the main interleukins responsible for the recruitment, activation, and accumulation of neutrophils. The interplay of both cytokines and their overproduction promotes multi-organ failure. According to the study conducted by Phetsouphanh, C., et al. [4], IL-8 levels were observed to be significant higher in subjects with LC. However, in our study, we did not find significant differences in IL-8 levels among our groups. Based on our results, it is possible to suggest that a basal inflammatory state predisposes individuals who contracted the SARS-CoV-2 infection to develop LC syndrome. It also positions these inflammatory cytokines as therapeutic targets in affected individuals. Multiple studies have demonstrated that elevated D-D levels are correlated with increased risks of sepsis and septic shock in patients. Increased D-D levels were notably more frequent in patients who had suffered severe SARS-CoV-2 infections requiring hospitalization when compared to patients with non-severe forms of the disease. In our current research, we assessed D-D concentrations in individuals who recovered from acute COVID-19 infections. Our findings showed that the levels of D-D were higher in the non-sequalae LC and LC groups in comparison to the healthy donor group. However, the most significant difference was observed in the LC and MetS group. Although it has been proven that obesity, hypertension, and diabetes are risk factors for the development of severe COVID-19 and are associated with increased fatal outcomes [28], the information regarding patients with LC is limited. Our results confirmed that D-D values remain very high in patients with metabolic disorders and LC, even months after the infection, which exacerbates the complications related to the underlying condition and increases the risk of thrombotic events. Evaluating D-dimer levels in the extended period following COVID-19 infection could potentially influence diagnostic and treatment strategies for patients who have recovered from COVID-19. On other hand, it is known that inflammatory and coagulation responses are influenced by various types of adhesion molecules and selectins. Among these, P-selectin has received special attention because of its interaction with the glycoprotein ligand P-selectin (PSGL-1) on leukocytes, promoting the formation of platelet–leukocyte aggregates, and the release of procoagulant microvesicles [29,30]. P-selectin is also involved in platelet–platelet aggregation, which plays a significant role in inmunothrombosis. Our results showed significative increases in the group with LC and MetS, emphasizing the significance of closely monitoring patients with metabolic diseases as increases in markers such as P-selectin and PSGL-1 raise the risks of developing atherosclerotic and vascular lesions. Furthermore, endothelial damage triggers the release of anti-fibrinolytic mediators such as PAI-1, which, in turn, inhibits fibrinolysis, leading to the accumulation of fibrin within the intra-alveolar space. In a study conducted by Yu Zuo et al., increased PAI-1 levels were observed in patients with severe COVID-19 diagnoses. They reported that the persistence of fibrin was mediated by the overexpression of PAI-1, which overcame the local uPA and tissue-type plasminogen activator (tPA) [31]. Collectively, these alterations create an elevated hypercoagulable state, which is also observed in LC. Gorog D. et al. [32] reported that circulating soluble CD40L levels were significantly increased in patients with COVID-19 compared to healthy controls, suggesting a relationship with our results regarding the presence of persistent COVID symptoms and inflammatory disease progression. On the other hand, P-selectin on platelets interacts with PSGL-1 on leukocytes, promoting the formation of platelet and leukocyte aggregates, inducing the release of procoagulant and proinflammatory microparticles, thereby facilitating the positive regulation of various leukocyte cytokines. A recent study examined elevated levels of TF in the bloodstream and the likelihood of mortality among COVID-19 patients in intensive care units. Tissue factor (TF) is a glycoprotein found within cell membranes and is widely recognized as a key regulator of blood clotting, primarily safeguarding essential organs susceptible to physical damage. Moreover, TF acts as a cell surface receptor, creating a complex with the coagulation factors VII and VIIa. This complex initiates the activation of factor IX (FIX) and factor X (FX), setting off a series of reactions that produce thrombin and fibrin and activate platelets, ultimately leading to the formation of hemostatic clots at injury sites. Therefore, our results confirmed the presence of procoagulant activity in patients with LC, and this activity is exacerbated when underlying conditions such as MetS are present. The virus invasion of endothelial cells could potentially lead to inflammation and injury, resulting in the release of prothrombotic agents such as vWF, which is stored within Weibel–Palade storage bodies, and the exposure of the underlying collagen to which vWF binds. In previous studies, we confirmed that COVID-19 patients exhibited significantly elevated levels of vWF in their plasma samples, which was directly correlated with the severity of the disease. Recently, Fogarty et al. [21] demonstrated that convalescent COVID-19 patients had high concentrations of vWF in their plasma samples, similar to what has been observed in acute cases of this disease.

Our results showed the implications of an active immunothrombotic state in the persistence of sequelae in subjects who had recovered from COVID-19, which were enhanced in patients with MetS. Given the population characteristics of Mexico, where a high percentage of subjects present with this syndrome, it is of the utmost importance to identify this population group and provide clinical follow-up given their predisposition for developing LC and the health risks associated with this syndrome.

## 4. Materials and Methods

### 4.1. Participants

A cohort of 68 volunteers with positive diagnoses of SARS-CoV-2 infection, confirmed by laboratory tests (PCR-RT), was included and classified as follows: non-sequelae COVID-19 (*n* = 19), long COVID (LC) (*n* = 23), and long COVID and metabolic syndrome (LC-MetS) (*n* = 26). Samples from 23 participants were included as healthy donors, and the samples from this group had been obtained from August to December 2019 as control groups for other studies and stored in our biobank before the COVID-19 pandemic, and so no SARS-CoV-2 infections were possible in them.

### 4.2. Samples

Whole human blood was obtained by clean venipuncture after signing the informed consent form. Blood for the plasma samples was collected in vacutainer tubes containing 3.2% sodium citrate anticoagulant (Becton Dickinson, Lakes, NJ, USA) and processed within 2 h after collection. The plasma samples were obtained by centrifugation at 3500 rpm for 15 min at room temperature and then stored at −70 °C until their use for biomarker assessments.

### 4.3. Immunothrombotic Biomarkers

The following biomarkers were analyzed: interleukin 6 (IL-6), interleukin 8 (IL-8), P-selectin (P-sel), P-selectin glycoprotein ligand 1 (PSGL-1), soluble CD40 ligand (sCD40L), type 1 plasminogen activator inhibitor (PAI-1), D-dimer (D-D), tissue factor (TF), tissular plasminogen (tPA), and coagulation factor IX (FIX) were analyzed in the plasma samples by flow cytometry using the LEGENDplex Kit™ (San Diego, CA, USA). Human Thrombosis Panel Standard from BioLegend^®^ following the instructions suggested by the supplier. Briefly, the plasma samples were incubated with beads that differed in their sizes and internal fluorescence intensities. Each set of beads was conjugated with an antibody specific for each analyte on its surface and acted as a capture bead for that analyte. After washing, a cocktail of detection antibodies was added, and each specific antibody bonded to its specific analyte, forming a sandwich between the capture-analyte-antibody beads. Subsequently, streptavidin-phycoerythrin (SA-PE) was added and the samples were taken to the CytoFLEX Beckman Coulter^®^ (Brea, CA, USA) equipment for analysis.

### 4.4. Von Willebrand Factor

The von Willebrand factor was analyzed in the plasma samples using a BIOMEDICA DIAGNOSTICS IMUBIND^®^ vWF ELISA kit (Windsor, NS, Canada). This method employs a goat polyclonal antibody as the capturing agent. The samples were placed in micro-test wells that had been pre-coated, and a polyclonal antibody conjugated with horseradish peroxidase (HRP) was employed to detect the vWF antigen that had bound to it. The introduction of perborate/3,3′,5,5′-tetramethylbenzidine (TMB) substrate and its subsequent interaction with the HRP produced a blue-colored solution. Ultimately, the assay was terminated by altering the pH. All assays were conducted in accordance with the specifications and recommendations provided by the supplier.

### 4.5. Statistical Analysis

The Shapiro–Wilk test was used for the normality tests. The data that followed normal distributions were expressed as means ± standard deviations. The comparison between groups was completed using the Kruskal–Wallis test. Additionally, the categorical variables were expressed in frequencies and percentages, whereas the differences between the groups were explored using a chi-squared test. All statistical tests were two-tailed, with the significance level set at *p* < 0.05. All data analyses were implemented using SPSS software version 24.0 (IBM Corporation, (Chicago, IL, USA), and GraphPad 6.0 software was used for the analysis (San Diego, CA, USA).

### 4.6. Ethics

This study was approved by the following appropriate institutional review board: the Ethics and Research Committee of the Faculty of Medical and Biological Sciences “Dr. Ignacio Chávez” Ethics Committee, with the registration number CEI/2022/X-292. Written informed consent was obtained from all participants from a legally authorized representative for their anonymized information to be published in this article.

## 5. Conclusions

Our findings demonstrated the correlation between an active immunothrombotic state and the persistence of post-COVID-19 symptomatology. They also proved that chronic inflammatory states, such as MetS, favor the perpetuation of an inflammatory process that is reflected in the propensity to present with LC. Although a majority of the population appears to recover completely after a SARS-CoV2 infection, there are patients that persistently present with increased plasma levels of both inflammatory and prothrombotic mediators. More research is needed to fully understand the clinical significance of these biomarkers in the context of LC.

## Figures and Tables

**Table 1:** Clinical and Demographic Characteristics of the Study Population.

Variable	Healthy Donors (n = 23)	Non-Sequelae COVID-19 (n = 19)	LC (n = 21)	LC-MetS (n = 26)	*p* Value
Age (mean ± SD)	25.4 ± 4.9	20.1 ± 2.4	21.7 ± 5.3	51 ± 15.1	0.007
Female (%)	70	84.2	61.9	53.8	0.185
BMI (mean ± SD)	24.9 ± 3.5	23.6 ± 3.1	21.9 ± 3.4	30.9 ± 1.8	0.081
Vaccinated against SARS-CoV-2 (%)	N/A	94.7	100	96.1	0.0605
Pfizer		84.2	85.9	50	
AstraZeneca		10.5	4.7	30.8	
CanSino		0	4.7	15.3	
Moderna		0	4.7	0	
Non vaccinated		5.3	0	3.9	

Comparisons between groups were conducted using ANOVA (Analysis of Variance) and Pearson’s Chi-squared test. BMI, body mass index.

**Table 2:** Frequency of symptoms presented in participants with LC and LC-MetS.

Characteristic	LC (n = 21)	LC-MetS (n = 26)	*p* Value
Headache	38.0	38.4	0.99
Tachycardia	9.52	3.8	0.57
Fever	4.7	3.8	0.99
Cough	61.9	34.6	0.08
Anxiety	47.6	26.9	0.22
Ageusia	4.7	7.6	0.99
Digestive problems	4.7	3.8	0.99
Fatigue	14.2	7.6	0.64
Anosmia	33.3	19.2	0.32
Myalgias	9.5	42.3	0.02
Sore throat	4.7	0	0.44
Low back pain	14.2	30.7	0.30
Arthralgia	14.2	38.4	0.10
Urticaria	9.5	3.8	0.57
Arrhythmia	4.7	0	0.45
Rhinorrhea	4.76	11.54	0.61
Sleep disorders	19.05	34.62	0.33

The results are expressed in percentage. Comparisons between groups were conducted using Pearson’s Chi-squared test.

## Data Availability

Data are contained within the article.
